# Supplementary figures and images for: Endophilin mediated endocytosis and epidermal growth factor receptor govern Japanese encephalitis virus entry and infection in neuronal cells
Source: PLoS Pathog. 2025 Dec 16;21(12):e1013790. doi: 10.1371/journal.ppat.1013790 (PMC12747170; doi:10.1371/journal.ppat.1013790)

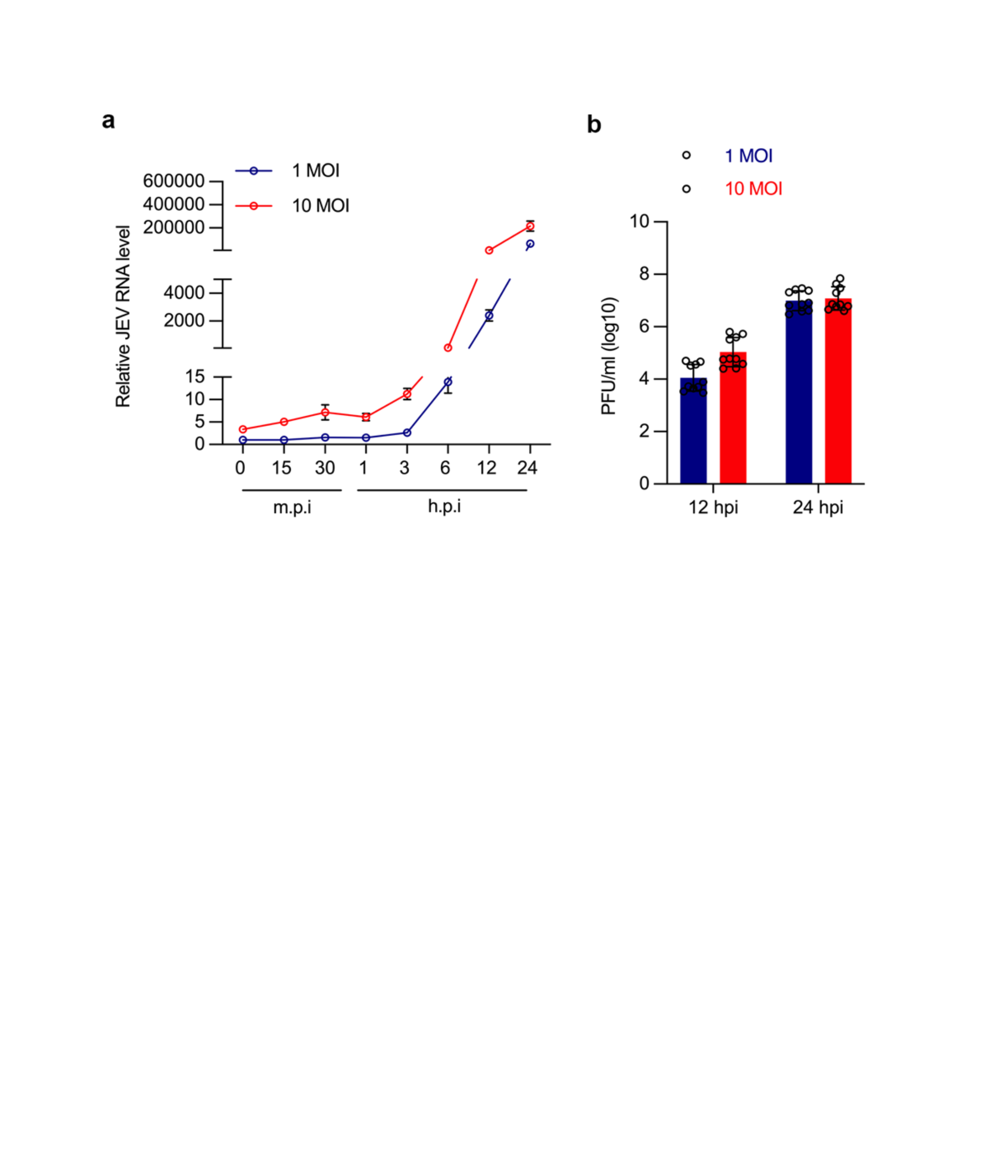

Supplement: S1 Fig — SH-SY5Y cells were infected with JEV MOIs 1 and 10 for 1 h and were harvested at 0 min, 15 min, 30 min, 1 h, 3 h, 6 h, 12 h, and 24 h post-infection. (a) The graph shows relative JEV RNA levels normalized to 0 mpi, MOI 1. (b) Viral titres at 12 and 24 hpi. All values are represented as mean ± S.D from at least two independent experiments. (TIF) [file ppat.1013790.s001.tif]

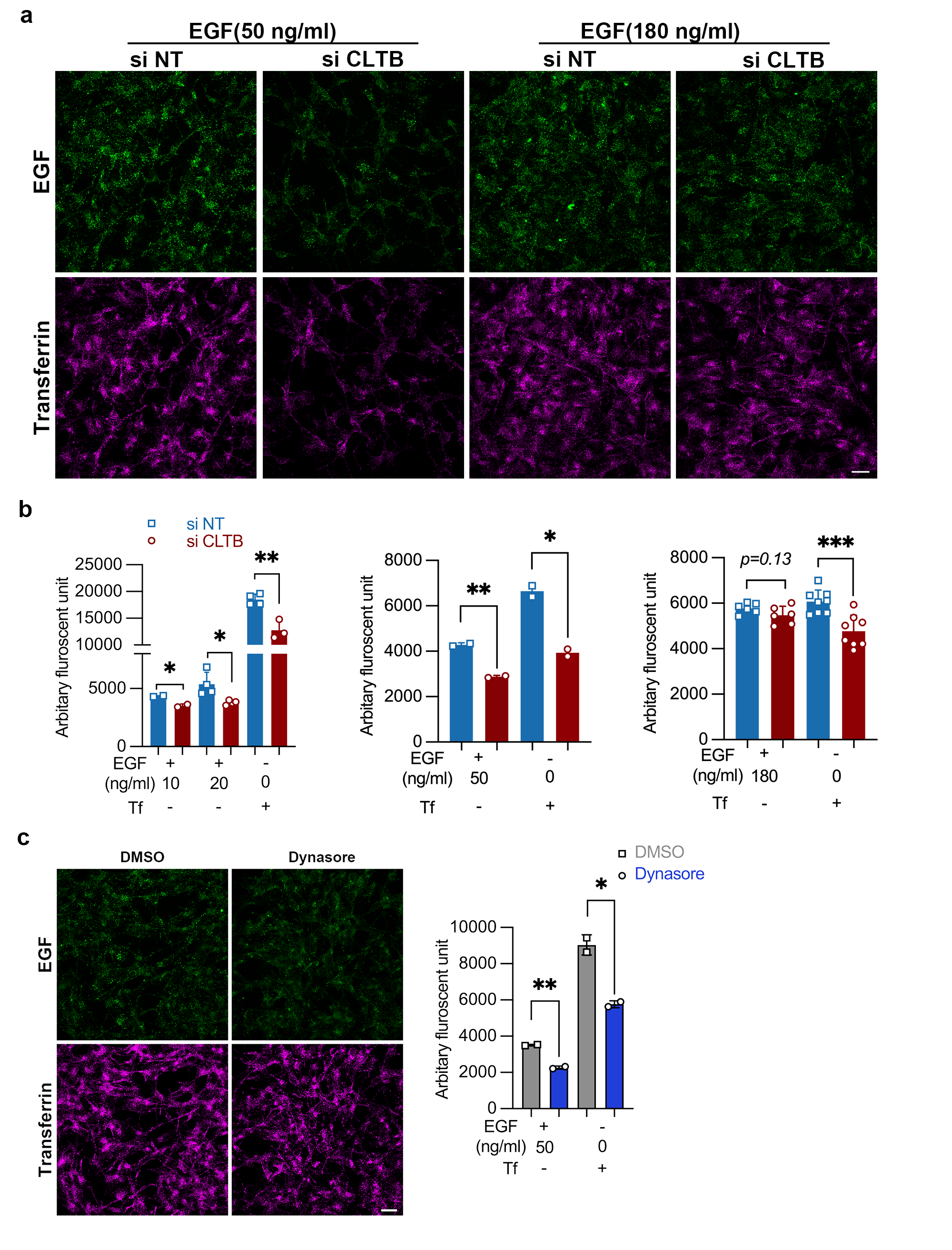

Supplement: S2 Fig — (a-b) SH-SY5Y cells transfected with siNT and siCLTB (72 h), were treated low (10, 20, 50 ng/ml), and high (180 ng/ml) concentration of Alexa fluor 555 EGF along with Alexa fluor 647 Tf (20 ng/ml) for 5 min at 37Oc. (a) Representative images indicating cargo uptake. Images were acquired using 63x objective, Scale: 20 µm. (b) Quantification of cargo uptake with a bar graph representing the total fluorescent intensities from 2 or more independent coverslips (~ 100 cells per coverslip). (c) SH-SY5Y cells seeded in Nu-serum containing media were pre-treated with either DMSO or 80 µM of dynasore for 1 h at 37Oc. Cells were then given a pulse of Alexa fluor 555 EGF (50 ng/ml) and Alexa fluor 647 Tf (20 ng/well) for 5 min at 37Oc. Images show uptake of EGF and Tf upon treatment with DMSO/dynasore. Scale, 20 µm. Bar graph compares the effect of dynasore treatment with DMSO control on the fluorescent intensities of EGF and Tf cargo. Total fluorescent intensities were quantified using Image J software with ~100 cells/coverslip. Individual values are representative of mean ± S.E.M. Statistical analysis was determined with student’s unpaired two-tailed t-test, NEJM: 0.12 (ns), 0.033 (*), 0.002(**), < 0.001(***). (TIF) [file ppat.1013790.s002.tif]

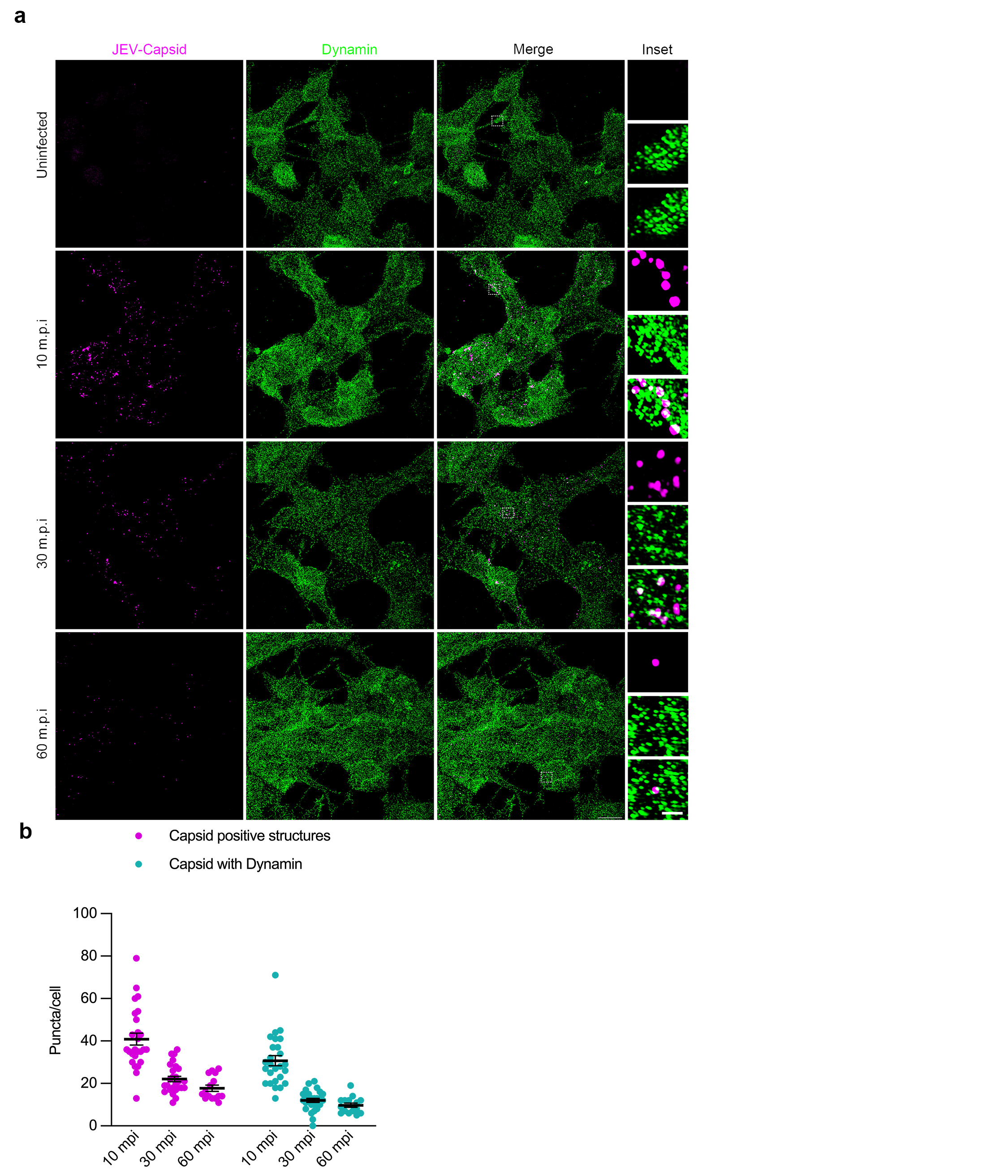

Supplement: S3 Fig — (a) SH-SY5Y cells were allowed to bind with 100 MOI virus on ice for 1 h and were subsequently shifted to 37Oc for 10 min, 30 min, and 60 min. Cells were fixed and immunostained for capsid (magenta) and dynamin (green), and then imaged using the Elyra PS1 (Carl Zeiss Super-resolution microscope). Insets show the magnified area from the confocal micrographs depicting the colocalization of dynamin puncta with capsid structures. Images are representative of two independent coverslips. Scale: 10μm, inset: 1μm. (b) The bar graph represents the quantification of the total number of capsid-positive structures and the total number of capsid structures colocalized with dynamin puncta per cell. All values are represented as mean ± S.E.M. (TIF) [file ppat.1013790.s003.tif]

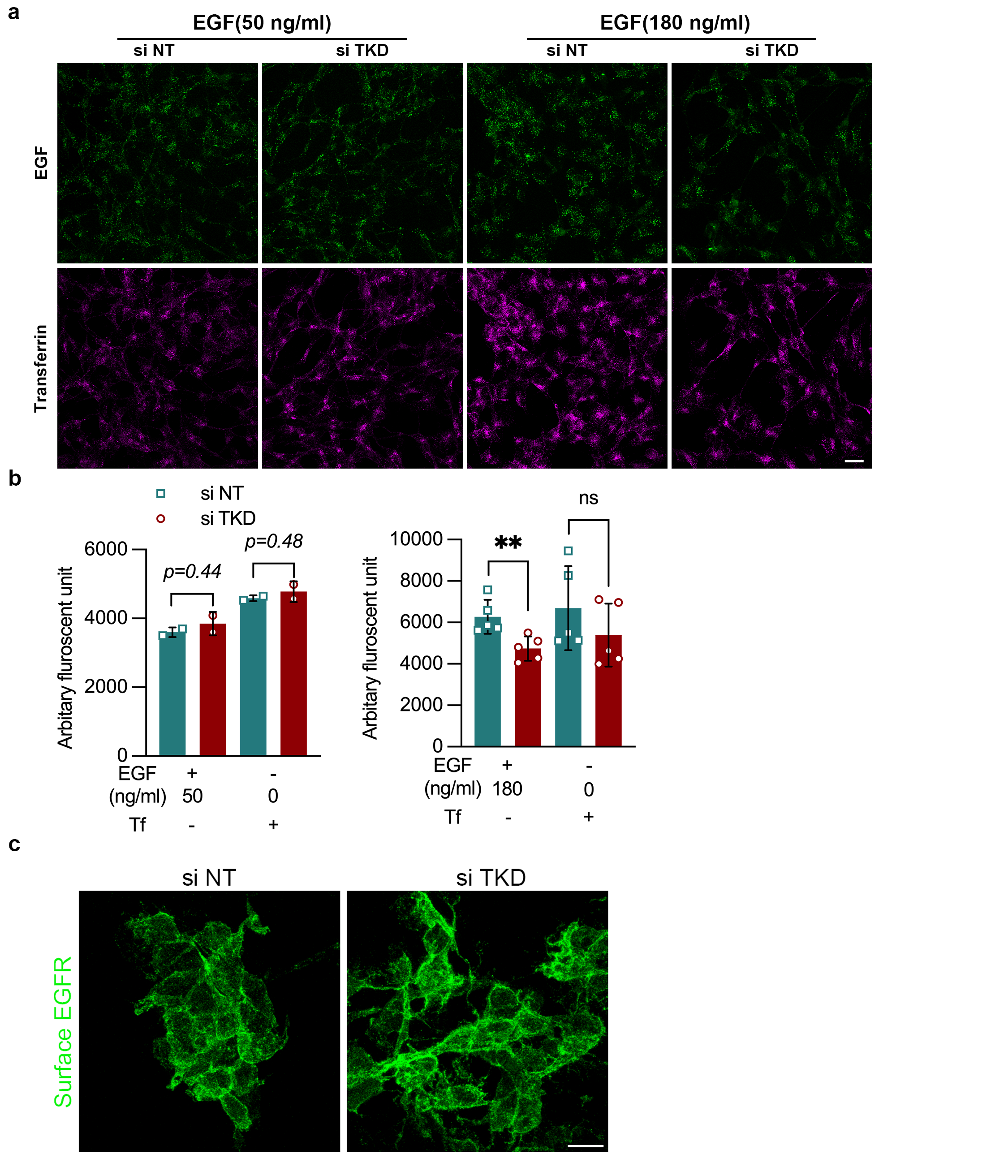

Supplement: S4 Fig — (a-b) SH-SY5Y cells transfected with siNT and three endophilin A1/A2/A3 siRNA: siTKD (72 h), were treated with Alexa fluor 555 EGF (50 & 180 ng/ml) and Alexa fluor 647 Tf (20 ng/well) for 5 min at 37Oc. (a) Representative images indicating cargo uptake. Scale, 20 µm. (b) The bar graph quantifies the total fluorescent units of EGF and Tf uptake under siNT and siTKD conditions. Analysis was performed using image J software with ~100 cells/coverslip, represented as mean ± S.E.M. Statistical analysis was determined with unpaired student’s t-test, NEJM: 0.12 (ns),0.033 (*), 0.002(**), < 0.001(***). (c) SH-SY5Y cells treated as above were stained for cell surface EGFR. A clear enhancement in the cell surface EGFR levels is observed siTKD cells; Scale: 10 μm. (TIF) [file ppat.1013790.s004.tif]

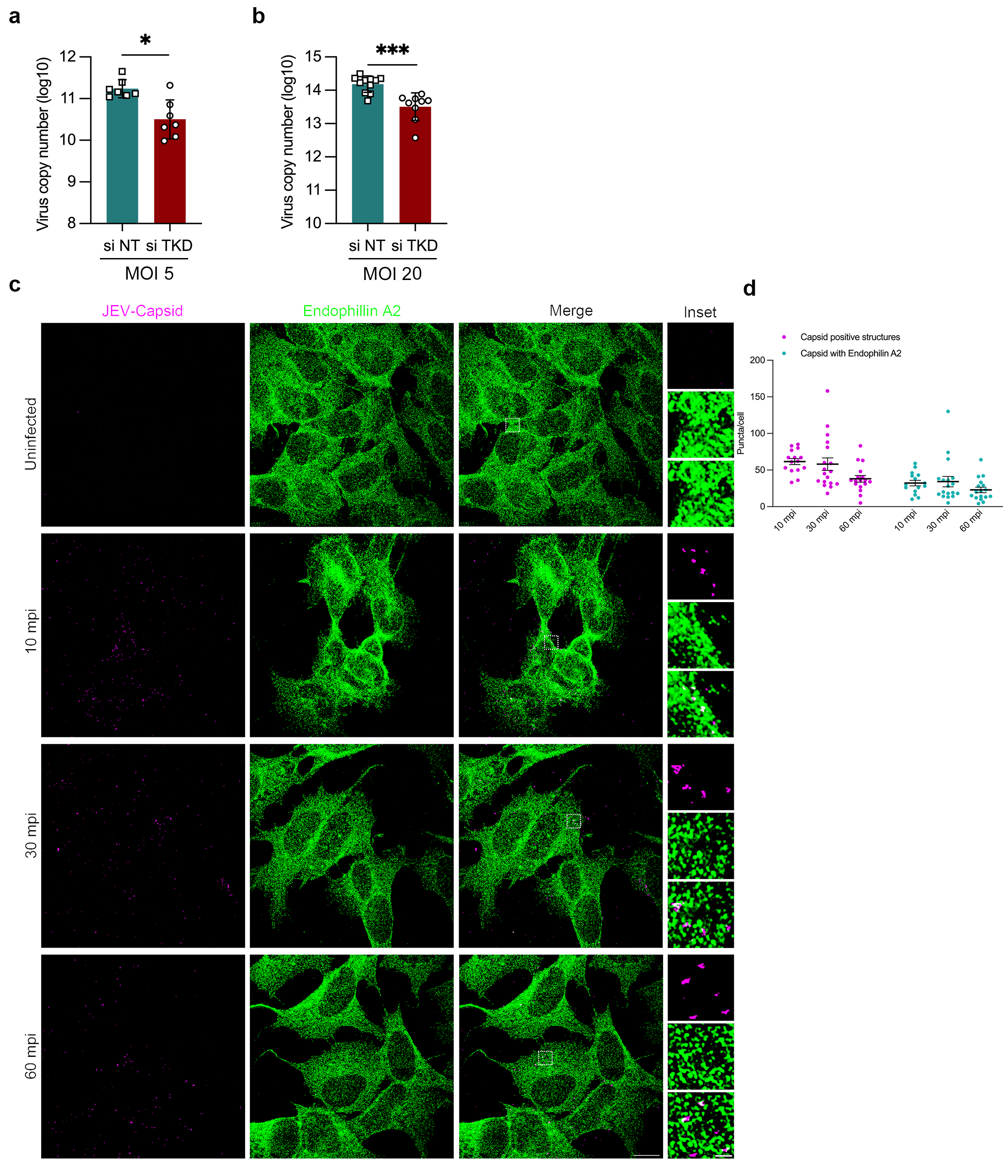

Supplement: S5 Fig — (a) SH-SY5Y cells were transfected with all three endophilin A1/A2/A3 siRNA (siTKD) for 72 h. Cells were infected with JEV MOI 5 & 20 at 37Oc for 1 h, and virus internalization assays were performed. Absolute virus envelope copies were detected with qRT-PCR. Data shown are from two or more independent experiments represented as mean ± S.D. Statistical analysis was determined with Mann Whitney test with 95% confidence level, NEJM: 0.12 (ns), 0.033 (*), 0.002(**), < 0.001(***). (b) SH-SY5Y cells were allowed to bind with 100 MOI virus on ice for 1 h and were subsequently shifted to 37Oc for 10 min, 30 min, and 60 min. Cells were immunostained for capsid (magenta) and endophilin A2 (green) and imaged on Elyra PS1 (Carl Zeiss Super-resolution microscope). Insets show the magnified area from the confocal micrographs depicting the colocalization of endophilin A2 with capsid structures. Images are representative of two independent experiments. Scale: 10μm, inset: 1μm. (c) The bar graph represents the quantification of the total number of capsid-positive structures and the total number of capsid structures colocalized with endophilin A2 per cell. All values are represented as mean ± S.E.M. (TIF) [file ppat.1013790.s005.tif]

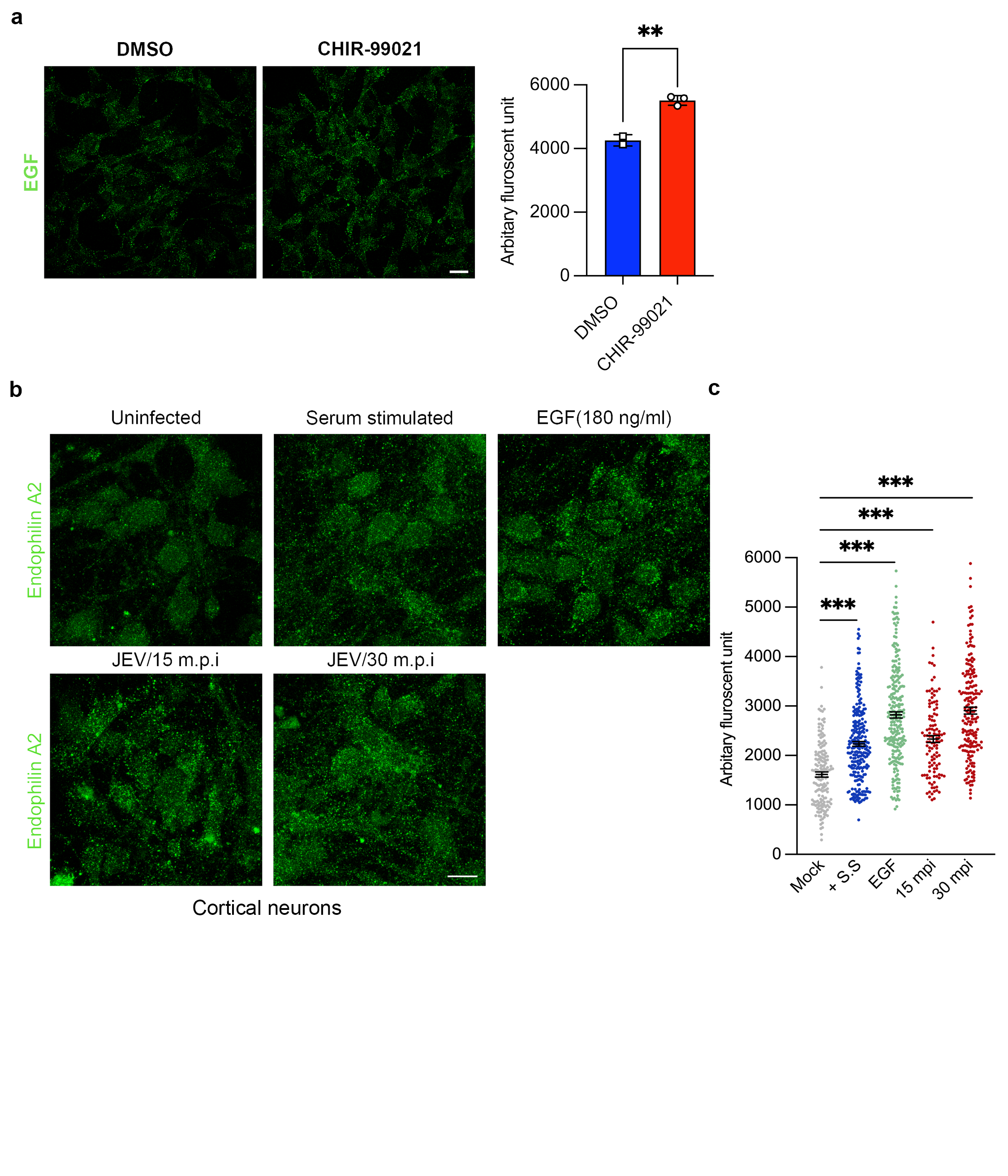

Supplement: S6 Fig — (a) SH-SY5Y cells were pre-treated with CHIR-99021 for 1 h at 37Oc and then pulsed with Alexa fluor 555 EGF (180 ng/ml) for 5 min at 37Oc. Cells were fixed, and images were acquired at a 63x objective Scale, 20 µm. Bar graph shows the quantification of the total fluorescent unit of EGF in DMSO and CHIR-99021 treated conditions. (b) Primary cortical neurons were left uninfected, 20% serum-stimulated, treated with EGF (180 ng/ml), or infected with JEV (100 MOI) for 15 min and 30 min respectively at 37Oc. Cells were fixed and stained with endophilin A2, and images were acquired using a 63x objective scale:10 µm. (c) The bar graph quantifies the total fluorescent unit of endophilin-positive puncta in different conditions. Analysis was performed using image J software with ~100 cells/coverslip. All values are represented as mean ± S.E.M; statistical analysis was determined with unpaired student’s t-test/Kruskal-Wallis test with Dunn’s multiple comparisons test. Statistical significance: NEJM: 0.12 (ns), 0.033 (*), 0.002(**), < 0.001(***). (TIF) [file ppat.1013790.s006.tif]

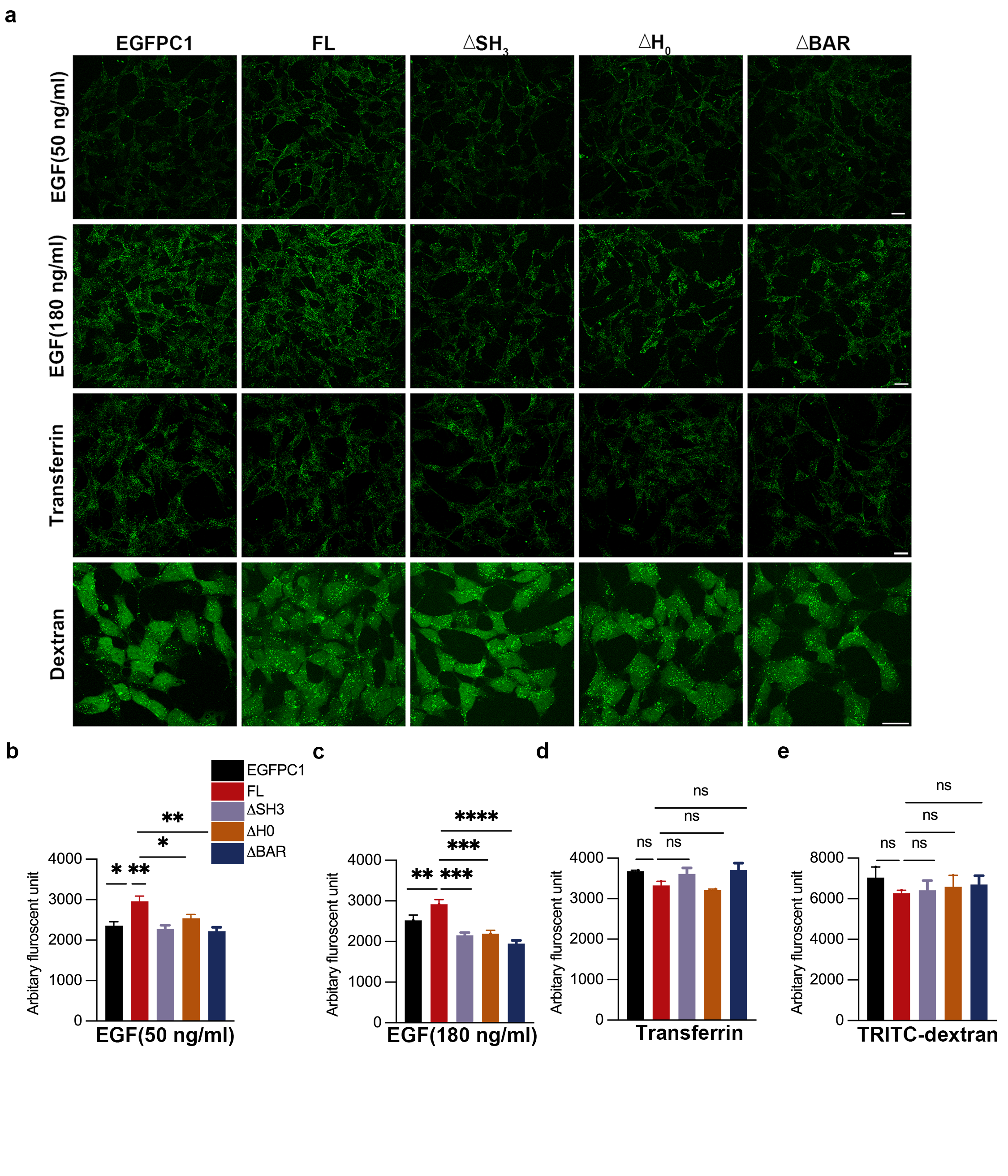

Supplement: S7 Fig — (a) SH-SY5Y cells with stable expression of EGFPC1 (empty vector), endophilin A full length (FL), truncated endophilin with ΔSH3, ΔH0, and ΔBAR domains were serum-stimulated (20% FBS), and pulsed with EGF-555 at 50 ng/ml or 180 ng/ ml for 5 min at 37Oc. For the Tf uptake assay, cells were serum starved for 30 min and were pulsed with 20 ng/well of Tf-568 for 5 min at 37Oc. Cells were pulsed with 200 ug/ml of TRITC- dextran (10,000 MW) for the fluid phase uptake assay for 10 min at 37Oc. Cells were fixed with 4% PFA, and images were acquired with 63x objective. Images are representative of the uptake of fluorescently labelled cargoes. (b-e) The bar graph represents the quantification of total fluorescent intensities. Analysis was performed using image J software from ~100 cells per coverslip. All values are represented as mean ± S.E.M; statistical analysis was determined with Ordinary one-way ANOVA with Dunnett’s multiple comparison test with 95% confidence level. (TIF) [file ppat.1013790.s007.tif]

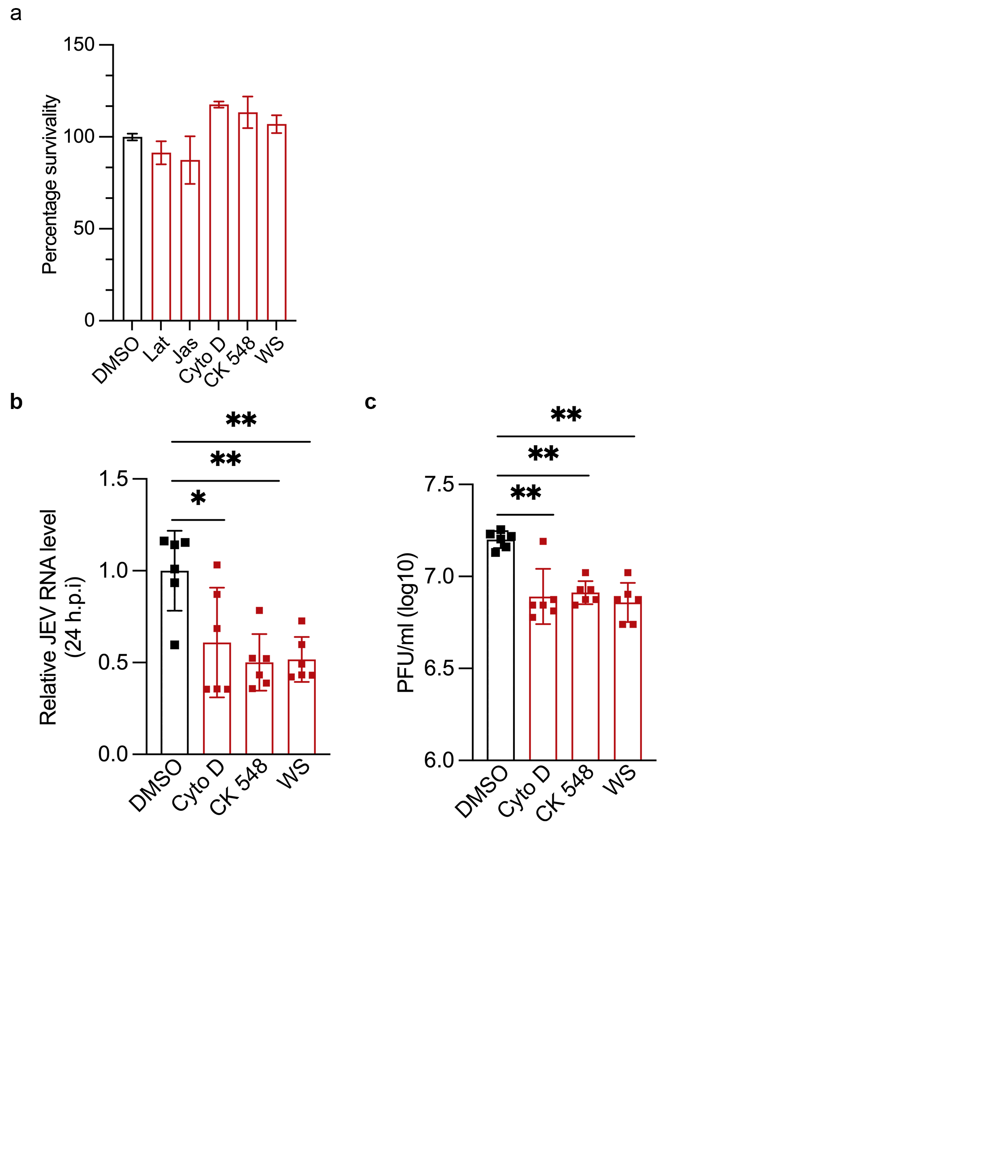

Supplement: S8 Fig — (a) SH-SY5Y cells were treated with the actin inhibitors LatA (1uM), Jas (1uM), CytoD (5uM), CK-548 (50uM) and Wiskostatin (10uM) for 4 h, and a cell viability assay was performed to establish non-toxic drug concentration. (b-c) Cells were pre-treated with DMSO control/ actin inhibitor for 1 h at 37Oc, infected with 1 MOI virus at 37Oc for 1 h, and harvested at 24 hpi. Viral RNA was determined by qRT-PCR (b), and the extracellular virus particles were detected with plaque assays (c). Data shown are from two or more independent experiments represented as mean ± S.D. Statistical analysis was determined by Ordinary one-way ANOVA with Dunnett’s multiple comparison test with 95% confidence level. Statistical significance: NEJM: 0.12 (ns),0.033 (*), 0.002(**), < 0.001(***). (TIF) [file ppat.1013790.s008.tif]

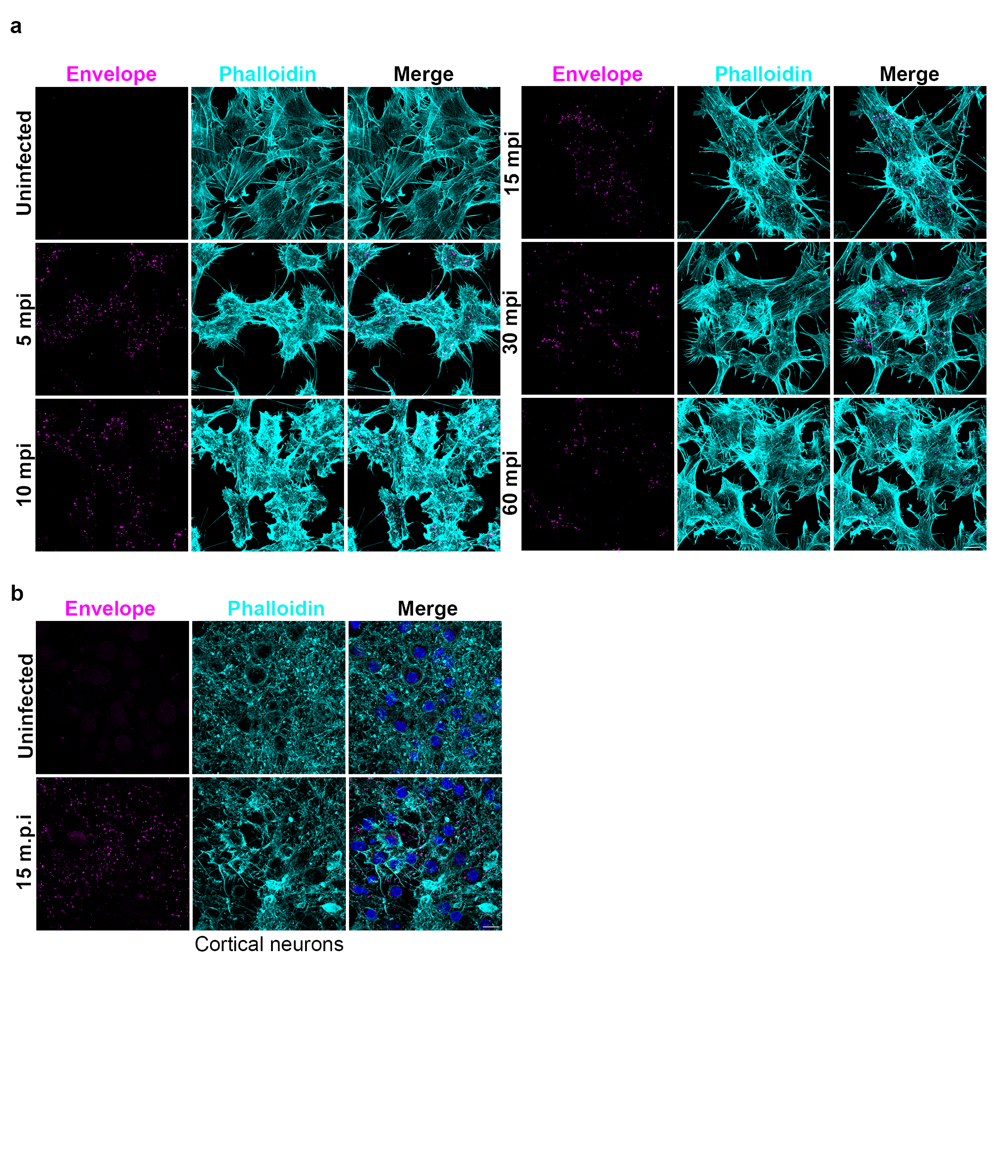

Supplement: S9 Fig — (a) SH-SY5Y cells were allowed to bind with 100 MOI JEV at 4Oc to allow attachment only and were subsequently shifted to 37Oc for internalization for 5-, 10-, 15-, 30-, 60- min. Cells were immunostained with JEV envelope antibody (magenta) and phalloidin (cyan) and imaged with Leica TCS SP8 microscope, 63x objective. The representative confocal micrograph shows the phalloidin-stained F-actin and JEV envelope puncta at the respective time points, scale: 10 µm. (b) Primary cortical neurons were infected with 100 MOI JEV at 4Oc to allow attachment only and were subsequently shifted to 37Oc for internalization for 15 min. Cells were fixed and immunostained with JEV envelope antibody (magenta) and phalloidin (cyan), nuclei with dapi (blue). The representative confocal micrograph shows the phalloidin-stained F-actin, envelope and nuclei in cortical neurons, scale: 10 µm. (TIF) [file ppat.1013790.s009.tif]

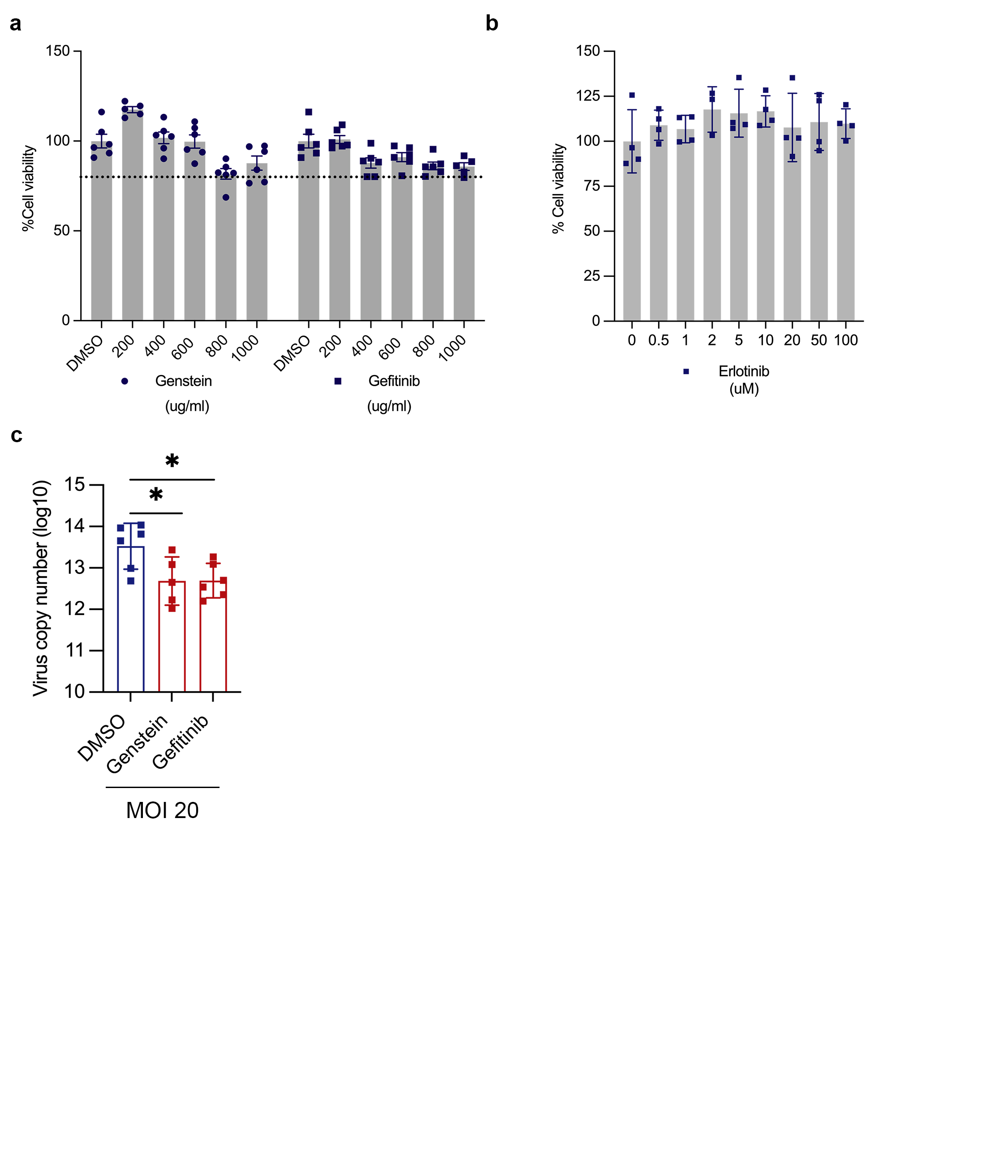

Supplement: S10 Fig — SH-SY5Y cells were either untreated or treated with genistein, gefitinib (a), or erlotinib (b) in a dose-dependent concentration for 5 h at 37Oc. MTT assay was performed to check the percentage of viable cells. Data shown are from two or more independent experiments represented as mean ± S.D. (c) Cells were serum starved overnight and treated with genistein (400 μg/ml) or gefitinib (400 μg/ml) for 5 h before infection. Virus entry assay was performed at 20 MOI, 4Oc, for 1 h and then shifted to 37Oc for 1h. Viral RNA was determined by qRT-PCR. All values are represented as mean ± S.D from at least two independent experiments. Statistical analysis was performed using ordinary one-way ANOVA, followed by Dunnett’s multiple comparison test with a 95% confidence level. Statistical significance: NEJM: 0.12 (ns),0.033 (*), 0.002(**), < 0.001(***). (TIF) [file ppat.1013790.s010.tif]

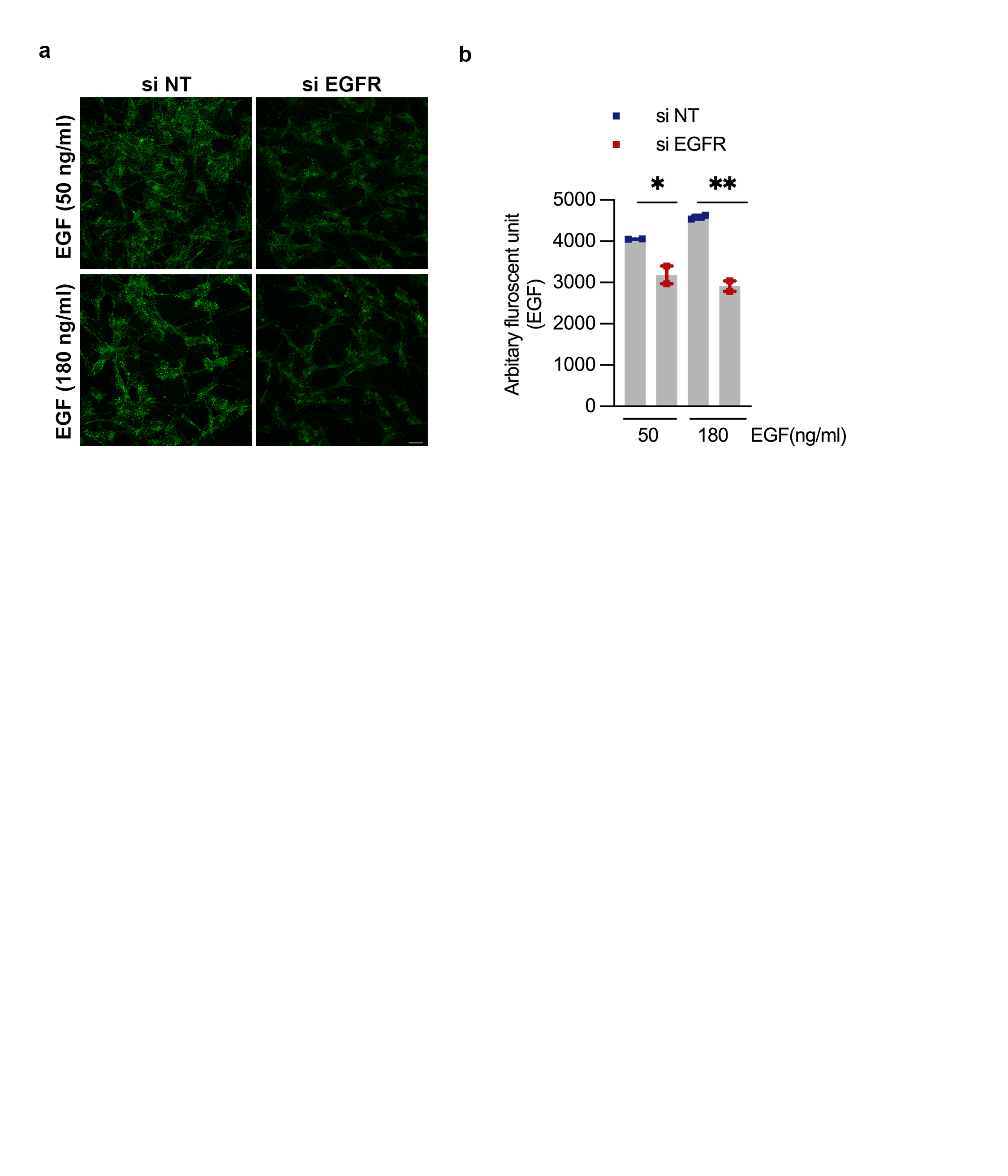

Supplement: S11 Fig — SH-SY5Y cells transfected with siNT/si EGFR for 72 h, were treated with Alexa fluor 555 EGF (50 & 180 ng/ml) for 5 min at 37Oc. (a) Representative images indicating cargo uptake. Scale, 20 µm. (b) Bar graph shows the quantification of the total fluorescent units of EGF uptake. Analysis was performed using image J software with ~100 cells/coverslip, represented as mean ± S.E.M. Statistical analysis was determined with Ordinary one-way ANOVA with Šídák’s multiple comparisons test, NEJM: 0.12 (ns),0.033 (*), 0.002(**), < 0.001(***). (TIF) [file ppat.1013790.s011.tif]

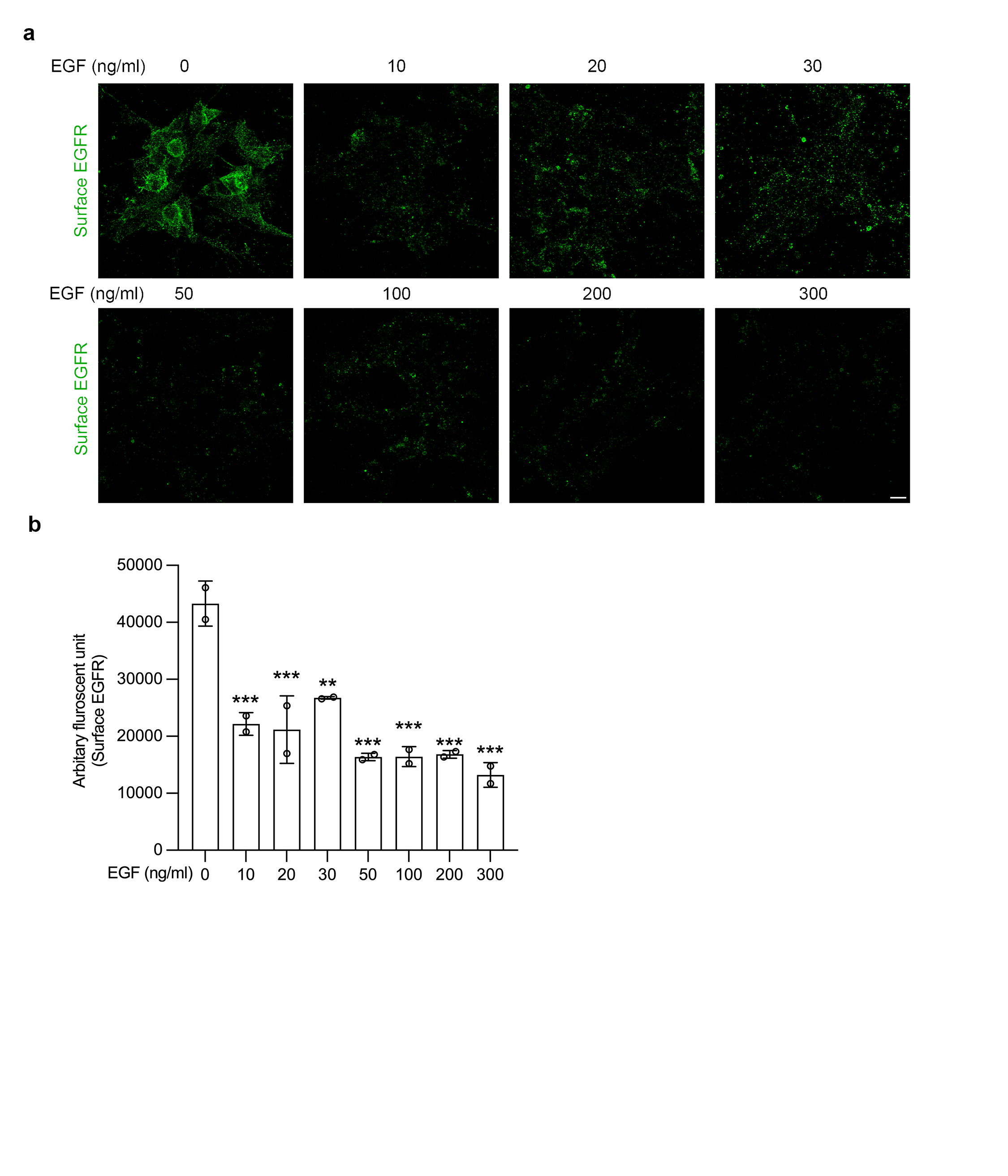

Supplement: S12 Fig — SH-SY5Y cells were serum stimulated with 20% FBS for 15 min at 37Oc followed by different doses of EGF stimulation (0, 10, 20, 30, 50, 100, 200, and 300 ng/ml) at 37Oc for 15 min. Cells were washed with PBS and labelled for cell surface EGFR. (a) Images show cell surface EGFR levels (green). (b) The bar graph shows the quantification of total fluorescent intensities, as measured using ImageJ software with ~100 cells/coverslip; scale: 10μm. Individual values are represented as the mean ± S.E.M. Statistical analysis was performed using ordinary one-way ANOVA with Dunnett’s multiple comparison test at a 95% confidence level. Statistical significance: NEJM: 0.12 (ns),0.033 (*), 0.002(**), < 0.001(***). (TIF) [file ppat.1013790.s012.tif]

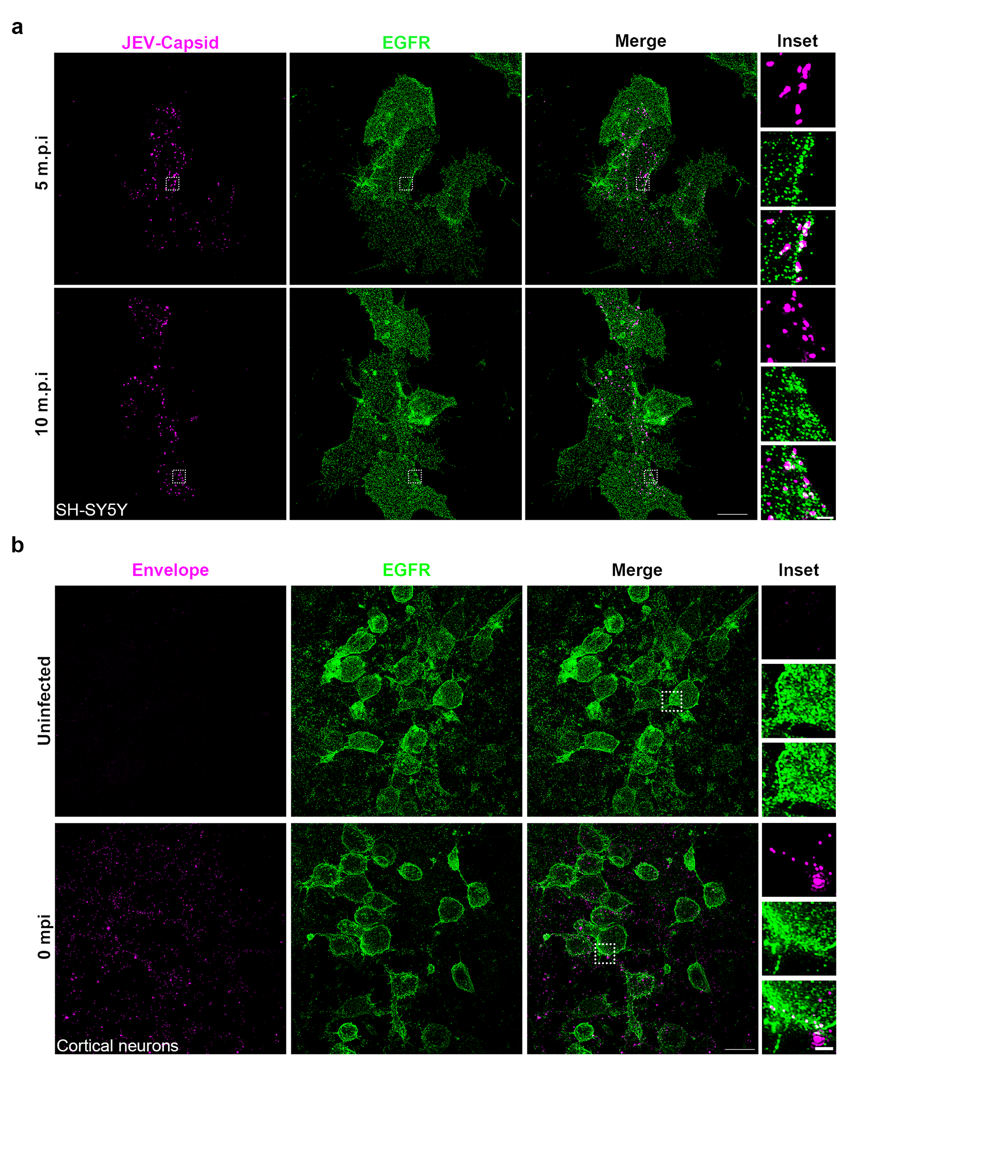

Supplement: S13 Fig — (a) SH-SY5Y cells were allowed to bind with 100 MOI of virus for 1 h on ice and were fixed post 5- and 10-min internalization at 37Oc. Cells were immunostained with JEV capsid (magenta) and EGFR (green) antibodies. Images were acquired in Elyra PS1 (Carl Zeiss Super-resolution microscope) using 63 x objective. Insets show the zoomed image of capsid-positive structures colocalized with EGFR; scale: 10 µm, inset: 1 µm. (b) Primary cortical neurons were incubated with 100 MOI of virus for 1 h on ice and were subsequently fixed post 0-, 10-, 30-, and 60- min of virus internalization. Cells were immunostained with JEV envelope (magenta), and EGFR (green) antibodies. Images were acquired with Elyra PS1 (Carl Zeiss Super-resolution microscope) using 63 x objective; insets show the zoomed image of envelope-positive structures colocalized with EGFR. Representative image with 0 min pi is shown, data for other time points not included here; scale: 10 µm, inset: 1 µm. (TIF) [file ppat.1013790.s013.tif]

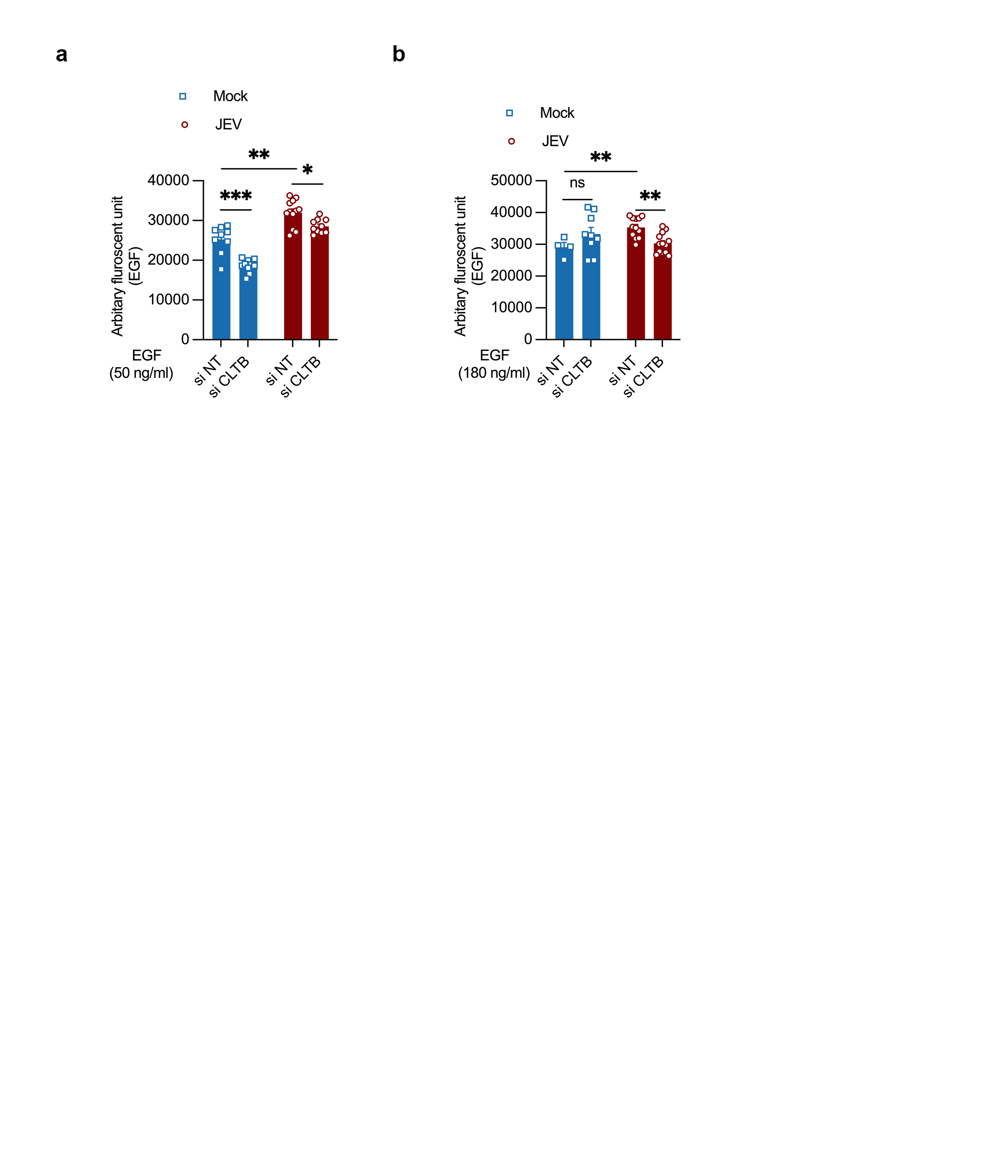

Supplement: S14 Fig — SH-SY5Y cells grown on coverslips were transfected with siNT and si CLTB for 72 h. Cells were mock-infected and JEV (MOI 1)-infected for 1 h and were pulsed with two different concentrations of Alexa Fluor 555 EGF (50 & 180 ng/ml) for 5 min at 37 °C. The bar graph quantifies the total fluorescent units of EGF uptake under siNT and siCLTB conditions. Analysis was performed using ImageJ software, represented as mean ± S.E.M. Statistical analysis was determined with unpaired Student’s t-test, NEJM: 0.12 (ns),0.033 (*), 0.002(**), < 0.001(***). (TIF) [file ppat.1013790.s014.tif]
