# Supplementary material for: Endophilin mediated endocytosis and epidermal growth factor receptor govern Japanese encephalitis virus entry and infection in neuronal cells
Source: PLoS Pathog. 2025 Dec 16;21(12):e1013790. doi: 10.1371/journal.ppat.1013790 (PMC12747170; doi:10.1371/journal.ppat.1013790)
Supplement: S1 Table — (PDF) [file ppat.1013790.s015.pdf]

S1 Table : List of chemical reagents used the study

S. No.	Name of Reagent	Catalogue No.
1.	Agarose Type VII	Sigma (A4018)
2.	BCA assay kit	G-Biosciences (786-570)
3.	Deoxyribonuclease I (DNase I)	SRL (61824)
4.	Dimethyl sulfoxide (DMSO)	Sigma (276855-250ML)
5.	EDTA	Sigma (E9884-500G)
6.	Glucose	Sigma (G8270-100G)
7.	HBSS	Gibco (14175095)
8.	HEPES	Sigma (H3375-25G)
9.	ImProm-II Reverse Transcription System	Promega (A3800)
10.	Luminol	Santa Cruz sc-2048
11.	MTT 3-(4,5-Dimethylthiazol-2-yl)-2,5-Diphenyltetrazolium Bromide	VWR life science, (0793-1G)
12.	Phenylmethylsulfonyl fluoride (PMSF)	Sigma (329-98-6)
13.	Poly (ethylene glycol) (PEG 400)	Sigma (202398-500G)
14.	Premix Ex Taq (Probe qPCR)	Takara (RR390A)
15.	ProLong Gold Antifade Mountant with DAPI	Invitrogen (P36935)
16.	Protease inhibitor cocktail (PI)	Sigma (P8340)
17.	Puromycin	InvivoGen (ant-pr-1)
18.	PVDF membrane	Merck Millipore (IPVH00010)
19.	PhusionTM High-Fidelity DNA Polymerase	Thermo scientific (F530S)
20.	QIAquick PCR &Gel Cleanup kit	Qiagen (28506)
**S. No.**	**Name of Reagent**	**Catalogue No.**
21.	Random hexamer	Sigma (H0268)
22.	SDS	Sigma (L3771-500G)
23.	Sodium pyruvate	HiMedia (TCL015)
24.	SYBR Premix Ex Taq	Takara (RR420A)
25.	Triton X-100	Sigma (T9284-500ML)
26.	TRIzol reagent (RNAiso Plus)	Takara (9109)
27.	Tween 20	G-Biosciences (RC1227)
**Media & other additives**		
28.	2XMEM	HiMedia (AL178A-500ML)
29.	B-27	Gibco (17504044)
30.	DMEM	HiMedia (AL007A-500ML)
31.	FBS	HiMedia, (RM10432-500ML)
32.	Geneticin Selective Antibiotic (G418 Sulfate)	Gibco (10131035)
33.	HiGlutaXL Dulbecco's Modified Eagle Medium	HiMedia (AL007G-500ML)
34.	L-15	HiMedia (AL011S-500ML)
35.	L-Glutamine	HiMedia (TCL012)

36.	MEM	HyClone (SH3024401-500ML)
37.	Neurobasal	Gibco (21103049)
38.	Penicillin-Streptomycin	HiMedia (A007-100ML)
39.	Trypsin - EDTA Solution	HiMedia (TCL007)
**Antibodies**		
40.	Chicken anti-Rabbit IgG (H+L) Cross-Adsorbed Secondary Antibody, Alexa Fluor 488, 1 mg	Invitrogen, A-21441
41.	Goat anti-Rabbit IgG (H+L) Highly Cross-Adsorbed Secondary Antibody, Alexa Fluor 647, 1 mg	Invitrogen, A-21245
42.	Donkey anti-Mouse IgG (H+L) Highly Cross-Adsorbed Secondary Antibody, Alexa Fluor 568, 1mg	Invitrogen, A10037
43.	Chicken anti-Mouse IgG (H+L) Cross-Adsorbed Secondary Antibody, Alexa Fluor 488, 1 mg	Invitrogen, A-21200
44.	Goat anti-Rabbit IgG (H+L) Cross-Adsorbed Secondary Antibody, Alexa Fluor 568	Invitrogen, A-11011
45.	Goat anti-Mouse IgG (H+L) Cross-Adsorbed Secondary Antibody, Alexa Fluor 647	Invitrogen, A-21235
46.	Alexa Fluor 555 EGF complex	Invitrogen (E35350),
47.	Alexa Fluor 568 conjugate transferrin	Invitrogen (T23365)
48.	Alexa Fluor 647 conjugate transferrin	Invitrogen (T23366)
49.	Alexa Fluor 546 phalloidin	Invitrogen (A22283)
50.	GAPDH	GeneTex (GTX100118)
51.	JEV core protein C	GeneTex (GTX131368)
52.	Dynamin Antibody (E-11)	Santa Cruz (sc-17807)
53.	Clathrin LC (D-21)	Santa Cruz (sc-32518)
54.	Endophilin I	Santa cruz (sc-134329)
55.	Endophilin II	Santa cruz (sc-365704)
56.	Endophilin III	Santa cruz(sc-376592)
57.	Clathrin light chain	Abcam (ab129326),
58.	EGFR	Santa cruz (sc-120)
59.	EGFR	CST, 2232
60.	Phospho-EGF Receptor (Tyr1068)	CST, 2234
61.	Akt	CST, 9272
62.	Phospho-Akt (Ser473)	CST, 9271
63.	Peroxidase AffiniPure Donkey Anti-Mouse IgG (H+L)	Jackson ImmunoResearch (715-035-150)
64.	Peroxidase AffiniPure Donkey Anti-Rabbit IgG (H+L)	Jackson ImmunoResearch (711-035-152)

65.	GFP	CST (2555)
66.	JEV-Envelope	Abcam (ab41671)
**Proteins**		
67.	Human EGF	Sigma (E9644)
68.	Recombinant Human EGFR Protein (ECD, hFc Tag)	Sino Biological, 10001-H02H
69.	Recombinant Human EGFR Protein (Isoform Viii, hFc Tag)	Sino Biological, 29662-H02B
**siRNA/Transfection reagents**		
70.	DharmaFECT 1	T-2001-03
71.	Lipofectamine RNAimax	Invitrogen (13778030)
72.	Lipofectamine 2000	Invitrogen (11668019)
73.	ON-TARGET plus Non-targeting (NT)	Dharmacon (D-001810-10-20)
74.	ON-TARGET plus human SH3GL1	Dharmacon (L-019582-00-0005)
75.	ON-TARGET plus human SH3GL2	Dharmacon (L-012597-00-0005)
76.	ON-TARGET plus human SH3GL3	Dharmacon (L-015728-02-0005)
77.	ON-TARGET plus human CLTB	Dharmacon (L-004003-00-0010)
78.	ON-TARGET plus human EGFR	Dharmacon (L-003114-00-0005)
**Small inhibitors**		
79.	Erlotinib	HY-50896
80.	Genistein	92136-10MG
81.	Gefitinib	SML1657-10MG
82.	Cetuximab	Selleckchem, A2000
83.	Dynasore	D7693
84.	CHIR-99021	4423
85.	Cytochalasin D (CytoD)	Sigma (CytoD, C8273)
86.	Latrunculin A (Lat A)	Sigma (Lat A, L5163)
87.	Jasplankinolide (Jas)	Sigma (Jas, J4530)
88.	CK 548	Sigma (C7499)
