# Supplementary material for: Endophilin mediated endocytosis and epidermal growth factor receptor govern Japanese encephalitis virus entry and infection in neuronal cells
Source: PLoS Pathog. 2025 Dec 16;21(12):e1013790. doi: 10.1371/journal.ppat.1013790 (PMC12747170; doi:10.1371/journal.ppat.1013790)
Supplement: S2 Table — (a) List of primer sequences for qRT-PCR. (b) Primer sequences used for cloning. (PDF) [file ppat.1013790.s016.pdf]

S2 Table: Primer details

(a) List of primer sequences for qRT-PCR

Genes	Forward (5'-3') sequence	Reverse (5'-3') sequence
hGAPDH	TGCACCACCAACTGCTTACG	GGCATGGACTGTGGTCATGAG
JEV	AGAGCACCAAGGGAATGAAATAGT TaqMan probe: CCACGCCACTCGACCCATAGACTG (5′ end FAM, 3′end TAMRA).	AATAAGTTGTAGTTGGGCACTCTG
JEV Envelope region	TTACTCAGCGCAAGTAGGAGCGTCTCAAG	ATGCCGTGCTTGAGGGGGACG
SH3GL1	GGGCAAGATCCCCGATGAG	CACCTGCTCGATGTCAGTCTC
SH3GL2	CCAAACCTTCAGGTGTCCAAA	GCATCCCCTCATACCAGTTCTC
SH3GL3	TTGGCTGTGTTCATAGAGGCA	TCGCATCTGTAGCTTGCTCTG
CLTB	CGAGGAGGCTTTCGTGAAGG	GCAGGCGGGACACATCTTT
EGFR	AGGCACGAGTAAGCTCAC	ATGAGGACATAACCAGCCACC

(b) Primer sequences used for cloning

S. No.	Name	Sequence
1.	FL-F	ATATAGATCTTCGGTGGCGGGGCTGAAG
2.	FL-R	ATATGAATTCTCACTGCGGCAGGGGC
3.	ΔH_O-F	ATATAGATCTGGAGGGGCCGAGGGGAC
4.	ΔH_O-R	ATATGAATTCTCACTGCGGCAGGGGCA
5.	ΔSH_3-F	ATATAGATCTTCGGTGGCGGGGCTGAAG
6.	ΔSH_3-R	ATATGAATTCTCACTGGTCCAGGGGC
7.	ΔBAR_1-F	ATATAGATCTATGTCGGTGGCGGGGC
8.	ΔBAR_1-R	CGCTTAGGGCGTGAGGAGGCCCCTCCGA
9.	ΔBAR_2-F	GTCGGAGGGGCCTCCTCACGCCCTAA
10.	ΔBAR_2-R	ATATGAATTCTCACTGCGGCAGGGGCACA
